# The Stance4Health Project: Evaluating a Smart Personalised Nutrition Service for Gut Microbiota Modulation in Normal- and Overweight Adults and Children with Obesity, Gluten-Related Disorders or Allergy/Intolerance to Cow’s Milk

**DOI:** 10.3390/foods11101480

**Published:** 2022-05-19

**Authors:** Marika Dello Russo, Paola Russo, José Ángel Rufián-Henares, Daniel Hinojosa-Nogueira, Sergio Pérez-Burillo, Silvia Pastoriza de la Cueva, Sascha Rohn, Alexandra Fatouros, Konstantinos Douros, Verónica González-Vigil, David Epstein, M. Pilar Francino, Alfonso Siani, Fabio Lauria

**Affiliations:** 1 Institute of Food Sciences, National Research Council, 83100 Avellino, Italy; mdellorusso@isa.cnr.it (M.D.R.); prusso@isa.cnr.it (P.R.); asiani@isa.cnr.it (A.S.); 2 Departamento de Nutrición y Bromatología, Instituto de Nutrición y Tecnología de los Alimentos, Centro de Investigación Biomédica, Universidad de Granada, 18071 Granada, Spain; jarufian@ugr.es (J.Á.R.-H.); dhinojosa@ugr.es (D.H.-N.); spburillo@ugr.es (S.P.-B.); spdelacueva@ugr.es (S.P.d.l.C.); 3 Instituto de Investigación Biosanitaria ibs. GRANADA, Universidad de Granada, 18071 Granada, Spain; 4 Department of Food Chemistry and Analysis, Institute of Food Technology and Food Chemistry, Technische Universität Berlin, 13355 Berlin, Germany; rohn@tu-berlin.de (S.R.); a.urbisch@tu-berlin.de (A.F.); 5 Institute of Food Chemistry, Hamburg School of Food Science, University of Hamburg, 20146 Hamburg, Germany; 6 Pediatric Allergy and Respiratory Unit, 3rd Department of Pediatrics, “Attikon” University Hospital, School of Medicine, National and Kapodistrian University of Athens, 12462 Athens, Greece; costasdouros@gmail.com; 7 Gestión de Salud y Nutrición S.L., 33003 Oviedo, Spain; vgonzalez@gsnsoft.es; 8 Department of Applied Economics, University of Granada, 18071 Granada, Spain; davidepstein@go.ugr.es; 9 Area de Genòmica i Salut, Fundació per al Foment de la Investigació Sanitària i Biomèdica de la Comunitat Valenciana (FISABIO-Salut Pública), 46020 Valencia, Spain; mpfrancino@gmail.com; 10 CIBER en Epidemiología y Salud Pública, 28001 Madrid, Spain

**Keywords:** gut microbiota, personalized nutrition, Stance4Health, i-Diet Stance4Health, overweight, obesity, coeliac disease, food allergies, adults, children

## Abstract

Unhealthy diets represent a major risk for the pathogenesis of metabolic and chronic inflammatory diseases. Improving the quality of diet is important to prevent chronic diseases, and diet-induced modifications of the gut microbiota (GM) community likely play an important role. The EU-funded Stance4Health project aims at performing a randomized clinical trial based on a nutritional intervention program in the context of normal weight and overweight adults as well as children with obesity and gluten-related disorders or allergy/intolerance to cow’s milk. The trial will evaluate the efficacy of a Smart Personalised Nutrition (SPN) service in modifying GM composition and metabolic function and improving consumer empowerment through technology adoption.

## 1. Introduction

Worldwide, almost one in three people does not have access to an adequate diet and suffers from at least one form of malnutrition: undernutrition, overweight or even obesity, and diet-related non-communicable diseases (DR-NCDs) [1]. In the last decades, the industrial handling of foods and the excessive consumption of processed and ultra-processed foods have led to an increase in the consumption of refined sugars and fats, decreasing the amount of dietary fibre [2]. This profound dietary simplification is likely capable of influencing gut microbiota (GM) diversity with consequent unexpected effects on immune and inflammatory responses [1,3]. Mammalian GM has recently been shown to play a role in the pathogenesis of “civilization diseases” such as obesity, cancer, cardiovascular disease, and diabetes [4,5], but also immune disorders such as coeliac disease [6] and food allergy [7]. GM plays a role in the regulation of the host’s energy balance. It enhances the efficiency of the energy harvest from foods and influences the synthesis, bioavailability, and function of nutrients. This activity produces different beneficial metabolites and microbial compounds such as short-chain fatty acids, metabolites of aromatic compounds, and neuro-active chemical substances, acting as signalling molecules for metabolically active organs [8,9].

The close relationship between GM and diet-related diseases suggests focusing research attention on evaluating their interactions to improve human health by modulating the diet [10].

Overweight and obesity, which have nearly tripled globally since 1975, are linked to more deaths worldwide than underweight [11]. Coeliac disease has been increasing worldwide. In Europe, the United States, and Australia, estimates of coeliac disease prevalence range from 1:80 to 1:300 children [12]. IgE-mediated food allergy affects up to 10% of the European population, and the prevalence is higher among children than among adults [13]. It represents a growing and threatening public health concern that can significantly impact the quality of life of children and their families [14]. In this context, a personalized approach based on differential responses to foods or nutrients that takes into account different genotype or phenotype characteristics, eating behaviours, and personal preferences could be more effective in changing habits and improving health status than a conventional one-size-fits-all intervention.

The Stance4Health project aims at designing a randomized clinical trial that will be used to validate and evaluate the efficacy of a Smart Personalised Nutrition (SPN) service in modifying GM composition and metabolic function and improving consumer empowerment through a technology adoption. The trial will be implemented in the context of normal weight and overweight adults as well as children with obesity and gluten-related disorders or allergy/intolerance to cow’s milk. The SPN service will be delivered to consumers through the nutritional software mobile app i-Diet Stance4Health, a wearable wrist band, capable of measuring daily activity, and the tailored production and delivery of foods for individuals or specific groups of the population (i.e., healthy adults, people with food allergies, coeliac disease, obesity, and overweight).

## 2. Materials and Methods

### 2.1. Study Design

The multicentre single-blind, placebo-controlled, randomized trial will be conducted in Oviedo by the University of Granada, Spain, at the Technische Universität Berlin, Berlin, Germany, at the University General Hospital of Patras, Patras, Greece, at the University Hospital of Ioannina, Ioannina, Greece, and at the University General Hospital “Attikon”, Athens, Greece.

The study design follows the Standard Protocol Items: Recommendations for Interventional Trials (SPIRIT) guidelines. The study is registered with the ISRCTN registry (https://www.isrctn.com/ISRCTN63745549) (accessed on 18 March 2022).

### 2.2. Eligibility Criteria

Five study populations will be included in the study:

(1) Apparently healthy adult subjects (both sexes), aged 20–65 years, with a body mass index (BMI) between 20–28 kg/m^2^, stable weight, ability to use a smartphone, and Internet connection availability. Exclusion criteria are the diagnosis of chronic gastrointestinal (GI) disorders, coeliac disease, or chronic diseases such as diabetes or other metabolic diseases; present pregnancy or lactation (<6 weeks before study start), or intention to become pregnant in the next 12 weeks; recent inflammation and/or long-term use of an-ti-inflammatory drugs; medically prescribed diet or specific dietary regimens for any reasons (i.e., high-protein diet, vegetarianism, veganism, etc.); antibiotic treatment (<3 months before study start); intake of antioxidant, pre- or probiotic supplements (<1 month before study start); intense physical activity (>10 h/week); alcohol consumption >21 drinks/week for men and >14 drinks/week for women.
(2) Normal weight children (of both sexes), aged 6–11 years, with a BMI >5th and <85th percentile for age, gender, and height. Exclusion criteria are the diagnosis of chronic GI disorders and any other chronic disease, elimination diet, intake of probiotics (<2 weeks before study start).
(3) Children (of both sexes) with obesity, aged 6–11 years, with a BMI ≥95th percentile for age, gender, and height. Exclusion criteria are the diagnosis of chronic GI disorders and endocrinopathies, intake of probiotics (<2 weeks before study start).
(4) Children (of both sexes) with gluten-related disorders, aged 6–11 years, diagnosed with coeliac disease or wheat allergy (sIgE), on elimination diet. The exclusion criteria are the absence of symptoms.
(5) Children (of both sexes) with allergy/intolerance to cow’s milk, aged 6–11 years, with an IgE-mediated milk allergy on an elimination diet from infancy, lactose intolerance (symptoms plus breath hydrogen positive test), overgrown IgE-mediated milk allergy but current aversion for milk, or nondefinable phenotype (children who avoid milk but do not belong to any of the previous phenotypes). Exclusion criteria are the diagnosis of other GI comorbidities.

### 2.3. Intervention Study and Mobile App i-Diet Stance4Health

**Figure 1:**
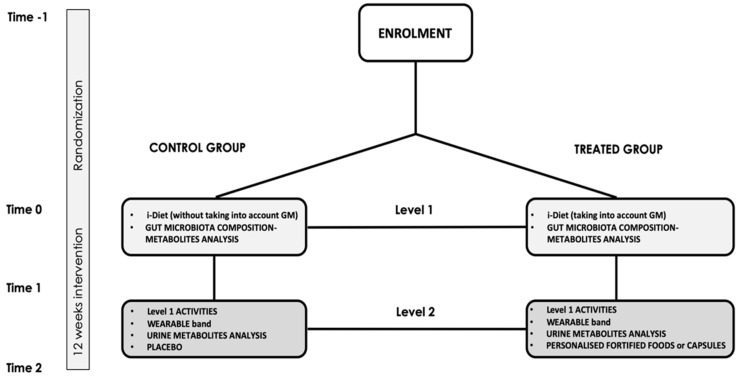
Intervention study.

This study will be a multicentre (five recruiting centres), parallel group randomised controlled trial, characterised by the use of the nutritional software mobile app i-Diet Stance4Health (Figure 1). A statistical model was developed to predict nutrients that must be incorporated into the diet of participants to transform their gut microbiota back into a healthy state, close to that of normal weight subjects. Based on the statistical model, the i-Diet Stance4Health app will suggest food choices in retailers and give participants specific advice about nutrition and lifestyle (taking into account or not GM composition, respectively for treated or control groups). The app will generate personalised diets (breakfast, lunch, dinner, snacks) based on Mediterranean dietary patterns encouraging fruits, vegetables, and whole grains consumption which are foods rich in compounds that have been shown to have positive effects on GM composition [15]. Tannins pills will supplement the diet since they have proven effective in modifying GM composition and functionality [16]. Participants’ health status, objectives, and habits (desired daily intakes, dishes that make up these intakes) will also be considered. The i-Diet Stance4Health will be used as a novel tool to record food intake (dietary assessment). To do so, participants, or parents in the case of children, will learn how to use the app through specific instruction/training. Specifically, they will indicate which serving size they usually prefer by pointing to it in the set of photographs to facilitate the estimation of the portion size. The users will also have the opportunity to make variations in the proposed menu. The app will estimate menus taking into account the nutritional values of foods and recipes by using a food composition database specifically developed in the framework of the Stance4Health project [17]. After a 2-week run-in period, the eligible participants will be randomly assigned to a 12-week intervention with two different levels of personalised nutrition: Level 1 and Level 2.

Level 1 (entry-level) intervention will consist in the use of i-Diet Stance4Health and the analysis of GM and metabolites. Level 2 (advanced level) will consist of the use of i-Diet Stance4Health and the analysis of GM and metabolites (as in the entry level), plus the use of a wearable wrist band only in adults, a metabolomics test to check the presence of metabolism disorders [18,19], and the intake of tannins extracts in pills for adults and in biscuits for children (treated group) or placebo (control group). The wearable band will be used to record physical activity, heart rate, blood pressure, and body composition as well as to refine food intake. Tannin extracts will be produced and distributed looking for a deeper modulation of the GM [16]. All data recorded in i-Diet Stance4Health and by the wearable band and results of the analyses will be recorded in specific databases. In detail, 200 Spanish and German adults will follow the entry-level intervention, and 200 will follow the advanced level intervention, equally divided into control and treated groups. The same criteria will be used for 400 Greek children (100 normal weight, 100 with obesity, 100 with gluten-related disorders, and 100 with allergy/intolerance to cow’s milk). Among them, half will be included in Level 1 and half in the Level 2 intervention.

### 2.4. Participants Timeline

Each intervention period will last 3 months. At the first contact, information about potential participants’ age, sex, anthropometry, dietary habits, health status, and contact details will be collected to select people with the inclusion/exclusion criteria identified for each study population. For this purpose, people will be encouraged to fill in specific questionnaires and return them by hand, by email, or online. Eligible participants will be asked to fill out the informed consent plus a number of questionnaires on their lifestyle, bowel, and dietary habits. Data will be collected using paper or, due to the epidemiological situation produced by the COVID-19 pandemic, online questionnaires [20]. In the latter case, participants will receive a link to open and accept the informed consent and to proceed with the completion of the questionnaires. On the Stance4Health webpage (www.stance4health.com, accessed on 18 March 2022), three different links (buttons) for the different languages (Spanish, German, and Greek) are included to allow participants to fill out the questionnaires online.

Three clinical evaluations of the study populations will be performed: at the beginning (T0), after 6 weeks (T1), and at the end of the interventions (T2) (Table 1). At T0 and T2, health [(blood pressure, previous pathologies, anthropometric (height, weight, waist circumference, fat mass, muscle mass)], dietary (food frequency questionnaires—FFQ), lifestyle (smoking habits, physical activity and sleep patterns), and socioeconomic status information will be collected in specific datasheets. Two FFQs will be used in the study, the FFQ_IDEFICS/i.Family [21] and the FFQ_S4H specifically developed for the study of GM. GM composition and metabolomic profile, and SCFAs and bile acids levels in feces will be assessed three times: at T0, T1, and T2.

At the end of the intervention, participants will have the opportunity to continue using the technologies for free as an incentive not to withdraw from the study. Moreover, all participants can choose to use the full functionality of i-Diet Stance4Health and associated elements (such as GM analysis and the food supplements), even if they were included in the control group. This could be an indicator of the acceptability and utility of the intervention.

### 2.5. Outcome Measures

Variation in GM alpha diversity, which will characterise species diversity change in each individual due to the impact of the intervention, represents the primary end point [22,23]. This end point is determined by 16S rRNA gene-targeted sequencing of microbial DNA isolated from stool samples. For selected samples, shotgun metagenomic and meta-transcriptomic sequencing will be applied.

Besides the changes in GM composition, consumer empowerment through technology adoption and long-lasting adoption of a healthy and sustainable diet are additional relevant outcomes of the intervention study.

The study comprises a number of secondary outcomes including appetite regulation and food intake, anthropometry (body weight, waist circumference, and body composition), blood pressure, physical activity levels, short-chain fatty acids and bile acids in feces, and urine metabolomics.

### 2.6. Sample Size and Power Calculation

The sample size was determined to detect an anticipated change in GM alpha diversity of 25% from baseline between control and treated groups. Based on a previously published trial conducted to test the efficacy of personalized nutrition intervention on alpha diversity [24,25], in the case of healthy adult subjects a sample size of at least 200 in each intervention level, totalling 400 individuals, will allow us to obtain a statistical power of 80% (beta) and alpha = 0.05. In case of loss to follow-up, extra volunteers will be enrolled to compensate. Due to the lack of specific data for children, the above calculations were extrapolated to the children’s group.

### 2.7. Recruitment and Randomization

Adult male and female participants for the personalised nutrition intervention will be recruited thanks to the collaboration with companies working in the field of nutrition and dietetics, from existing databases, and using the German and Spanish Stance4Health Facebook pages. Moreover, advertisements in local newspapers or TV programs will be used, and flyers, handouts, and posters will be distributed and affixed. In the case of an insufficient number of recruited subjects, additional participants will be recruited through the involvement of the mayors of the cities/villages in which the intervention will take place. Children will be recruited from the clinics of the University Hospitals of Ioannina, Patras, and “Attikon”, Greece.

The random allocation sequence necessary to assign individuals to each of the two levels of intervention, and further to the “control” and “treated” groups, will be provided and managed by an investigator who will not take part in the participants’ recruitment.

The randomisation list will be concealed to the investigational site. It will be stored under lock and key until database closure. After receipt of the closed database, the randomisation allocation sequence will be sent to the survey centres for statistical analysis.

### 2.8. Blinding

Blinding of participants will be possible thanks to the use of a placebo instead of tannin pills and biscuits enriched in tannins. Blinding of trial personnel is not possible because of differences between the intervention levels.

Trial personnel who will enroll participants, data collectors, outcome assessors, and data analysts will be blinded to treatment allocation, and an employee outside of the research team will manage data in the computer on separate datasheets.

### 2.9. Data Collection

Follow-up assessments and data collection will be undertaken by trial personnel at the University of Granada in Spain, at Technische Universität Berlin, Berlin, Germany, and at the University Hospitals of Ioannina, Patras, and “Attikon” Greece.

### 2.10. Compliance

To ensure good compliance, participants will be given detailed instructions and training to learn how to use the nutritional app i.Diet Stance4Health. Moreover, the nutrition app is designed to generate notifications to remind users to log all meals or update the necessary data and to provide nutritional or physical activity tips and alerts when they deviate from healthy parameters. Finally, to increase consumer empowerment, periodic feedback of the collected data will be sent to participants (i.e., after each week deviation in energy intake, cholesterol, iron, and other nutrients will be launched by the app).

### 2.11. Anthropometric Measurements and Body Composition

Weight and height will be self-reported by adults. For the children, measures will be taken by the survey staff using the same scales and stadiometers in the three survey centres (KaWe Height Measure, KaWe Kirchner & Wilhelm, and Seca 880 Weight Scale, Seca Ltd., for height and weight measurements, respectively). Body mass index (BMI) will be calculated as weight (kilograms)/height (square meters). Body composition in adults will be determined with the supplied wearable band. In children, it will be estimated by dual-energy x-ray absorptiometry (Hologic QDR series Discovery W densitometer, Hologic, Bedford, MA, USA).

### 2.12. Blood Pressure and Heart Rate

For adults, the wristband will measure blood pressure and heart rate. For children, blood pressure will be measured using oscillometric measurement with automated blood pressure and pulsometer.

### 2.13. Stool Samples

Stool sample containers will be provided to the participants and will be collected at T0, T1, and T2. The following measurements will be performed: fecal microbiota profiles, targeted metabolomic profiles (SCFAs and bile acids).

### 2.14. Urine Samples

Urine sample containers will be provided to the participants and will be collected at T0. Targeted analysis of specific metabolites will be performed to detect potential metabolic pathologies [16,17].

### 2.15. Blood Samples

Blood samples will be collected only in children at T0 and T2. The following biochemical parameters will be measured in blood samples: total protein, albumin, lipid profile, urea, creatinine, alanine and aspartate aminotransferase, alkaline phosphatase, electrolytes, ferritin, serum calcium, and vitamin D.

### 2.16. Storage of Biological Specimens

Biological samples will be stored under appropriate conditions and used exclusively for research purposes upon the consent of the donor.

### 2.17. Data Management

Data will be recorded on electronic datasheets and kept in an anonymous form. Sensitive data will be kept entirely separate from other data under lock and key and accessible only to key research team members. A 5-digit participant-ID code will be assigned to each subject included in the study. The ID code will be built as follows: the first digit denotes the country of residence, the second digit denotes children or adults, the last three digits denote the subject. Each document or item related to each subject will be labelled with the respective ID code.

Specific strategies will be employed to improve data quality during data collection, including accurate recruitment, a structured protocol, the inclusion of a run-in period, the use of standardized procedures, and the possibility to complete questionnaires online.

### 2.18. Statistical Analysis

The primary outcome (alpha diversity) will be analysed within each group using paired comparison t-tests to evaluate whether the changes from baseline to 12 weeks will be statistically significant. The absolute change (value at baseline subtracted from value after intervention for each subject) will be estimated with independent t sample tests. ANOVA will be used to analyse taxonomic diversity data and to assess the association of the GM composition with the phenotypic characteristics of the study population. Sensitivity analyses will be undertaken based on the assumption that missing outcomes are the worst possible, or the best possible, in different randomization groups. A significance value *p* < 0.05 will be considered statistically significant. Alpha diversity calculations and statistical analyses will be performed using various packages in the R platform, including the Vegan library (v2.5-2; Oksanen et al., 2012).

### 2.19. Monitoring

The trial will be monitored regularly by the survey teams and the local trustee, using a specific checklist. Online data access will be restricted to trained staff with unique password-protected accounts. Adverse events associated with the three months smart personalized nutrition intervention will be collected, discussed, and solved among field staff and the local responsible person for the different surveys.

## 3. Results

The overall objective of Stance4Health is to promote the adoption of personalised nutrition based on the use of smart mobile technologies as well as tailored food production, which will optimize GM activity. A set of new instruments developed within the project will allow for the adoption of healthy, pleasant, and sustainable dietary patterns based on the principles of the Mediterranean Diet, while also encouraging citizen engagement for an improved understanding of a healthy lifestyle. The nutritional intervention proposed here will test the efficacy of the SPN service developed by the Stance4Health project in promoting a healthy and environment-friendly lifestyle. The SPN service will be delivered to consumers through different tools such as the nutritional mobile app i-Diet Stance4Health, wearable wrist bands to measure daily activity, and production and delivery of food supplements and fortified foods for individuals or specific groups of the population (e.g., people with allergies, obesity, etc.).

To date, no studies are available that evaluated the effects of a SPN service based on the use of a nutritional app. It will allow a more personalised service to achieve better use of time, which is a benefit for the nutrition specialists and for consumers/patients.

The trial results will be important to understanding if the SNP service proposed by Stance4Health will be capable of ameliorating the intestinal microbiota and empowering consumers to make healthy and sustainable dietary choices.

## Figures and Tables

**Table 1:** Schedule of enrollment, interventions, and assessments for participants.

		Study Period		
	Enrollment	Allocation	Middle of the Study	End of the Study
**PROTOCOL ACTIVITY**	Day −60 to 1	Day 0	Week 6	Week 12
**TIMEPOINT**	−T1	T0	T1	T2
**ENROLLMENT:**				
Eligibility screen	X			
Informed consent	X			
Demographic details	X			
Lifestyle questionnaire		X		X
FFQ_IDEFICS/i.Family		X		X
FFQ_S4H		X		X
Bowel habits questionnaire		X		X
Allocation		X		
**INTERVENTIONS:**				
Study populations—Level 1		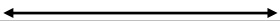		
Study populations—Level 2		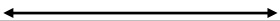		
**ASSESMENTS:**				
Nutritional app i.Diet		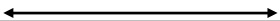		
Anthropometric measurements		X		X
Stool samples		X	X	X
Urine samples		X		

The arrows represent the duration of interventions/assesment. starting at “Day 0” and finishing at “Week 12”.
